# A model to facilitate the mental health of psychiatric nurses in a forensic unit to manage mental health care users’ hostile behaviour constructively

**DOI:** 10.4102/curationis.v41i1.1844

**Published:** 2018-06-13

**Authors:** Tebogo Tema, Marie Poggenpoel, Chris Myburgh

**Affiliations:** 1 Department of Nursing, University of Johannesburg, South Africa; 2 Department of Educational Psychology, University of Johannesburg, South Africa

## Abstract

**Background:**

Hostile behaviour by mental health care users (MHCUs) is prevalent in forensic units in South Africa, and this causes service providers distress and burnout. Psychiatric nurses (PNs) find it difficult to render quality care to MHCUs who are threatening them and also challenging their authority in a forensic unit. Forensic mental health care practitioners may be challenged to engage authentically with MHCUs who constitute a risk to their personal safety or who have committed acts the practitioner finds morally disturbing. There is a need to facilitate the mental health of PNs in a forensic unit to manage hostile behaviour constructively.

**Objective:**

The objective of this article is to describe the process that was followed in developing, implementing and evaluating a model that could be used as a framework of reference to facilitate the mental health of PNs in a forensic unit to manage hostile behaviour constructively.

**Method:**

A theory-generative, qualitative, exploratory descriptive and contextual study design was used to develop the model. The steps of the process entailed the identification of the central concept, the definition of the central concept and other essential criteria and the classification of the central and related concepts. The model was then described and evaluated.

**Results:**

The central concept was identified as the ‘facilitation of empowerment of PNs to manage hostility in a constructive manner’, defined, classified and then described and evaluated.

**Conclusion:**

The model as framework of reference could assist PNs in managing hostility in a forensic unit constructively.

## Introduction

The overall purpose of forensic care is to promote the quality of life of mental health care users (MHCUs) and enable their re-entry to a safe and healthy life in the community (Jeon, Gang & Oh 2017:94). The MHCUs have the right to safe nursing care and the nurse has a corresponding legal and moral responsibility to provide such care (Pera et al. 2011:148; Videbeck 2014:154). In psychiatric institutions in South Africa, some forensic units are full, and the MHCUs are cared for in prisons owing to a lack of space in the forensic units (Tema 2017:1). Mental health care users in a forensic unit often display hostility towards nurses who are caring for them (Tema 2010:33–46). Bekelepi, Martin and Chipps (2015:151) observed that aggression and violence by MHCUs towards psychiatric nurses (PNs) are global issues. Psychiatric nurses in a forensic unit have contact with people diagnosed with anti-social personality disorder (Pryjmachuk 2011:417), and anti-social personalities have been strongly associated with aggression and violence.

### Problem statement

Psychiatric nurses in a forensic unit at a psychiatric institution faced challenges to their mental health. These PNs experienced hostile behaviour from MHCUs, resulting in fear, disempowerment and emotional distress (Tema 2010:33–46). There seemed to be a gap in knowledge and practice regarding an approach to facilitate these PNs’ mental health. The research question arose: What could be done to facilitate the mental health of PNs who experienced hostile behaviour by MHCUs in a forensic unit at a psychiatric institution in the Limpopo province?

By developing a model to facilitate the mental health of PNs, they will be assisted to manage the hostile behaviour by MHCUs in a constructive manner.

### Research purpose

The purpose of the research study was to describe, implement and evaluate a model that can be used as a framework of reference for the advanced psychiatric nurse (APN) to facilitate the mental health of PNs in a forensic unit to manage hostile behaviour constructively.

### Research objectives

The research study objectives were to:

- derive central concepts to be utilised in a model to facilitate the mental health of PNs in a forensic unit to manage hostile behaviour constructively;
- describe relationships between these concepts;
- describe a model as a framework of reference for an APN;
- implement and evaluate the model.

### Definition of key concepts

The key concepts model, facilitation, mental health, manage, constructively and hostility were defined.

#### Model

A model is a symbolic representation of an empiric experience in the form of words, pictorial or graphic diagrams, mathematic notations or physical material (Chinn & Kramer 2011:157). In this study, a model is a graphic diagram representing a process whereby an APN facilitates the mental health of PNs to manage hostility by MHCUs constructively.

#### Facilitation

Facilitation is defined as a dynamic interactive process through the creation of a positive environment and mobilisation of resources, as well as the identification and bridging of obstacles in the promotion of health (University of Johannesburg 2012:7). Facilitation in this study is the process whereby the PNs who are experiencing hostile behaviour by MHCUs in a forensic unit are actively engaged in various activities by an APN. These activities enable them to acquire knowledge, skills and attitudes that are essential in facilitating their mental health, in order for them to manage hostility by MHCUs in a forensic unit in a constructive manner.

#### Mental health

Mental health is a dynamic interactive process that reflects harmonious interaction of the internal and external environments of the PN, as well as mobilisation of resources, to promote mental health as part of holistic health and well-being (University of Johannesburg 2012:5). In this study, mental health is the congruent integration of the physical, spiritual and psychosocial aspects of a PN, and access to available resources essential for enhancing mental health as a component of holistic health.

#### Manage

To manage is to carry out the task of ensuring that a number of activities are performed in such a way that a defined objective is achieved. To manage is to succeed in doing something difficult (Longman South African School Dictionary 2007:420). In this study, managing refers to the concerted efforts of the PNs in a forensic unit in the application of their knowledge, a wide range of skills and attitudes, to manage hostility by MHCUs constructively.

#### Constructively

Constructively means being useful or helpful, serving to build or improve (Oxford South African Concise Dictionary 2013:156). In this study, constructively refers to the ability of PNs in a forensic unit to manage hostility by MHCUs in a helpful manner, thus enhancing the mental health of PNs.

#### Hostility

Hostile behaviour is behaviour characterised by verbal abuse, threatening and aggressive behaviour, uncooperativeness and behaviours that have been defined as undesirable or in violation of established limits (Schultz & Videbeck 2005:74). In this study, hostility is behaviour characterised by lack of a constructive nurse–patient relationship, threats from MHCUs, ineffective communication and MHCUs challenging PNs’ authority.

## Research design and method

### Design

A theory-generative, qualitative, exploratory, descriptive and contextual design was utilised to develop and implement the model (Austin, Goble & Kelecevic 2009: 272; Burns & Grove 2009:51; Babbie 2013: 90,91; Babbie & Mouton 2009: 272; Chinn & Kramer 2011:216; Creswell 2014: 8; Grove, Gray & Burns 2013:66; Maree 2007:70; Walker & Avant 2011:7).

### Method

The four steps of model development were followed (Chinn & Kramer 2011:169–186). In step 1, concept analysis, the central concepts were identified from a field study, defined (Wandelt & Stewardt 1975:61–69) and classified (Dickoff, James & Wiedenbach 1968:343). In step 2, the relationships were described between the concepts (Walker & Avant 2011:176). The model was described in step 3 (Chinn & Kramer 2011:186) and in step 4 the model was evaluated (Chinn & Kramer 2011:185).

### Measures to ensure trustworthiness

Trustworthiness was observed throughout the study by using Guba’s model of trustworthiness criteria (Lincoln & Guba 1985:301–331). Kuckartz (2014:12) asserts that one of the most important reasons to proceed with methodical rigour when analysing data is trustworthiness. The criteria for trustworthiness entailed credibility, transferability, dependability and confirmability. The researcher used credibility by multiple strategies during data collection, and included interviews, observation, field notes and reflexive journal (Creswell 2009:191). The researcher presented the model structure and findings at research forums and doctoral seminars, and corrections and modifications were made based on the recommendations by experts in research. Dependability was ensured by providing an in-depth description of the steps followed in the entire study, supported by literature review. In this study, the supervisors continuously audited the study. Transferability was enhanced by providing a rich description of the demographics of participants and descriptions of findings as well. Confirmability refers to objectivity, that is, the potential for congruency between two or more independent people about the accuracy, relevance or meaning of the data (Polit & Beck 2014:323) and, therefore, the two supervisors and the independent coder worked closely with the researcher to ensure confirmability.

### Ethical consideration

In this study, ethical conduct was ensured by following ethical standards as set out by Dhai and McQuoid-Mason (2011:43), namely the principle of respect for autonomy, the principle of non-maleficence, the principle of beneficence and the principle of justice. The researcher considered ethical issues from the outset of the research project (Oliver 2004:15). Ethical clearance AEC40-01-2013 was obtained from the Academic Ethics Committee of the University of Johannesburg. Permission was also obtained from the chief executive officer of the particular psychiatric institution. The researcher treated participants with respect, as autonomous agents who have the right to make decisions regarding their participation in the research (Bless, Higson-Smith & Sithole 2014:30; Grove, Burns & Gray 2013:164). Information about the purpose of the study and the objectives were clearly explained to the participants, and participants fully understood the consequences of the agreement (Saks & Allop 2013:84; Streubert & Carpenter 2011:61). Participants were informed of any foreseeable emotional risk or discomfort that might be experienced during the participation in the study, and measures to minimise the risk were discussed (Fox & Bayat 2013:72; Picardi & Masick 2014:33). They ensured that anonymity was maintained by using fictitious names (LoBiondo & Haber 2014:259). The physical, mental, social and spiritual aspects of participants were respected (Weathington, Cunningham & Pittenger 2010:42). Justice was ensured by treating all the participants equally (Moule & Goodman 2014:59).

## Results

### Step one: Concept analysis

In this study, concept analysis was performed by exploring the PNs’ experience of hostility in a forensic unit by MHCUs. This was carried out in two phases, namely identification and definition of the central concept and the classification of concepts (Chinn & Kramer 2011:175–181).

### Step one: Concept identification

In the researchers’ previous study of the PNs’ experience of hostile behaviour by MHCUs in a forensic unit (Tema 2010:33–46, 2011:918–922), the findings of the study revealed that PNs in a forensic unit experienced lack of constructive nurse–patient relationships, fear related to threats from MHCUs and disempowerment associated with a lack of knowledge and support by management, which culminated in emotional distress, resulting in the use of coping mechanisms. Psychiatric nurses were desperate to have control in a forensic unit, and it became clear that ‘facilitation of empowerment’ of PNs was the central concept. See Table 1.

**TABLE 1:** Identification of central concept based on the results of field study on psychiatric nurses’ experiences of hostile behaviour by mental health care users in a forensic unit.

Themes	Identified central concepts
Experienced lack of constructive nurse–patient relationships	Facilitation of empowerment of PNs
Experienced fear related to threats from MHCUs	
Disempowerment associated with lack of knowledge and support by management	
Experienced emotional distress, resulting in the use of coping mechanisms	

*Source:* Tema, T.R., 2010, ‘Psychiatric nurse experience of hostile behaviour by patients in a forensic unit in an institution in Limpopo Province’, Minor dissertation, M Cur Psychiatric Nursing, University of Johannesburg, pp. 33–46

PN, psychiatric nurses; MHCU, mental health care users.

### Step two: Definition and classification of concepts

The concept ‘facilitation of empowerment was defined as a dynamic interactive process between the APN and PNs, making it possible for PNs in a forensic unit to have authority, by providing them support to help them to be competent in managing the hostility of MHCHs in a forensic unit in a constructive manner’. Competency by PNs in managing hostility in a constructive manner results in empowered PNs, which enhances the mental health of PNs.

The concepts were classified as follows:

The agent is the person who facilitates empowerment. In this study, the agent is the APN who enables the disempowered PNs in a forensic unit to manage hostility in a constructive manner.

The recipient is the person or persons who benefit from the facilitation of empowerment process. In this study, the recipients are the PNs who work in a forensic unit at this specific psychiatric institution and participate in the empowerment process.

The context is the environment in which the empowerment of PNs is facilitated. In this study, the context is a forensic unit, a special unit in a psychiatric institution that caters for MHCUs who had committed offences owing to their mental illness and were found not to be fit to stand trial.

The dynamics in the model entail PNs experiencing hostility by MHCUs in a forensic unit, leading to distress, feeling of incompetency, marginalisation, hopelessness, culminating in disempowerment.

The procedure is the process of facilitating the empowerment of PNs by the APN, enabling them to obtain authority, providing them with support, to be competent in managing hostility in a constructive manner. The process comprised three phases: the relationship-building phase, the working phase and the termination phase. Through the process, the APN makes it possible for the PNs to move from feeling disempowered to feeling empowered.

Terminus is the empowered PNs who manage hostility in a constructive manner (see Table 2).

**TABLE 2:** Thinking map for conceptual framework.

Survey list	Description
Agent	Advanced psychiatric nurse
Recipient	Psychiatric nurses
Dynamics	Lack of constructive nurse–patient relationships, fear related to threats from MHCUs, disempowerment associated with lack of knowledge and support by management, and emotional distress resulting in the use of coping mechanisms
Procedure	Facilitation of empowerment of psychiatric nurses: obtaining authority, support and competence
Context	Forensic unit in a psychiatric institution
Outcome	Empowered psychiatric nurses to manage hostile behaviour of MHCUs constructively

*Source*: Tema, T.R., 2017, ‘A model to facilitate the mental health of psychiatric nurses in a forensic unit to manage hostile behaviour constructively’, D Cur Psychiatric Nursing Science, University of Johannesburg, p. 87

MHCU, mental health care users.

### Step two: Relationship statements

In this study, the relationship statements were formulated as follows:

The facilitation process is initiated by the APN who engages the PNs in the forensic unit in a dynamic interactive process, thereby building a trusting relationship. The APN shows respect for the PNs as important persons with spirit, mind and body, to make it possible for them to express their feelings.

The APN enables the PNs to obtain authority, by giving them support, to make decisions independently with regard to managing hostility in a constructive manner. Psychiatric nurses are assisted to look at alternative ways of becoming competent in managing hostility in a constructive manner.

Competency by PNs in managing hostility in a constructive manner results in empowered PNs.

### Step three: Description of the model

#### Structure of the model

The description of the structure of the model is based on the following: theoretical definitions, relationship statements, purpose of the model, assumptions of the model and the process description. The theoretical definitions and the relationship statements have been discussed in the paragraphs above.

#### Purpose of the model

The purpose of the model is to provide a frame of reference that can be used by the APN to facilitate the empowerment of PNs in a forensic unit to manage hostility by MHCUs in a constructive manner.

#### Assumptions of the model

In this study, the assumptions of the model were adapted from the Theory of Health Promotion in Nursing of the Department of Nursing (University of Johannesburg 2012:4). The assumptions are as follows:

- The APN and PNs are seen holistically in integrated, harmoniously interaction with individuals in the forensic unit (Tema 2017:86).
- Both have internal environments that consist of body, mind and spirit. Three aspects are interrelated and interdependent, working as a system. The ultimate outcome of the facilitation of empowerment of PNs, that is, competency, will lead to the physical, mental and spiritual well-being of the PN (Tema 2017:86).
- The PN has an external environment that consists of physical aspect, such as the home or church, that provide comfort; social aspect, that is, colleagues, family and friends; and a spiritual aspect that entails divine connection with God (Tema 2017:86).
- Psychiatric nursing is an interactive dynamic process, whereby an APN facilitates the promotion of mental health of PNs. In this interactive process, there is a trusting relationship and mutual involvement between both parties in the facilitation of empowerment of the PNs (Tema 2017:87).
- The APN uses himself or herself in a therapeutic manner, conveying sensitivity as he or she applies his or her knowledge, wide range of skills and values to facilitate the empowerment of the PNs, which results in the promotion of their mental health (Tema 2017:87).
- The promotion of mental health implicates mobilisation of resources by the APN, in facilitating the empowerment of PNs (Tema 2017:87).
- Mental health is a dynamic, interactive process in PNs’ environment, the forensic unit (Tema 2017:87).
- The relative mental health of PNs is reflected by their interaction in the forensic unit, where they are able to manage hostility by MHCUs in a constructive manner, which is the ultimate goal (Tema 2017:87).

#### Process description

**FIGURE 1:**
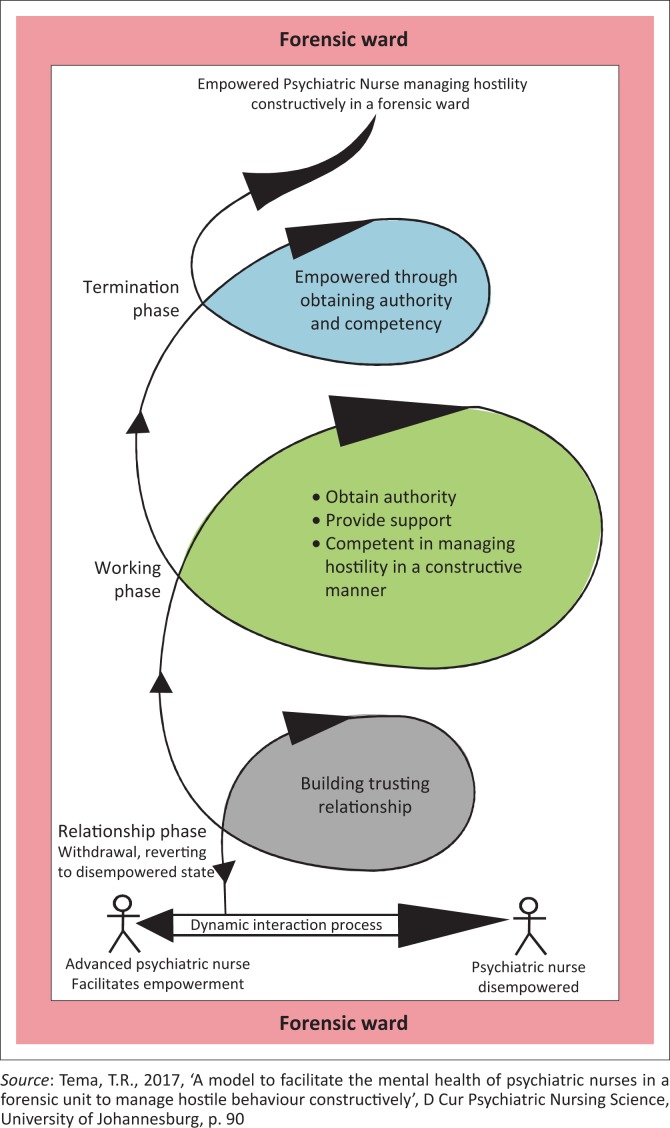
A model to facilitate the empowerment of psychiatric nurses to constructively manage hostility by mental health care users in a forensic unit.

The structure of the model is depicted in Figure 1. It has a base, three oval structures representing the phases of the model, and they are in a pink border, representing the forensic unit. The following is the progression of the model to facilitate the empowerment of PNs.

Phase 1 is the relationship-building phase and it has two aspects, that is, dynamic interactive process and making it possible. The dynamic interactive process serves as a foundation on which the APN lays the three phases of empowerment and also as a vehicle for the facilitation of the empowerment process. The APN conveys an attitude of acceptance that is essential in creating a warm and safe environment to allay PNs’ fears. The APN explains his or her role and the roles of the PNs, to work collaboratively. Making possible is the second aspect of the relationship-building phase, and in this session the focus is on assessment and identification of challenges. The APN engages the PNs in brainstorming to explore their purpose, objectives and expectations. The PNs make a commitment to participate actively in the empowerment process. James (2008:71) asserts that a commitment plan provides opportunity for follow-up checkpoints and motivational reminders.

The working phase entails three aspects: PNs having authority, providing support and helping them to be competent. The APN makes it possible for the PNs to have control over their situations, by encouraging them to exercise their rights to independence in making decisions and being responsible and accountable for the outcomes of the decisions in managing hostility in a constructive manner. The APN as a resource person applies his or her specialised knowledge, wide range of skills and attitude to provide emotional and informational support to the PNs and bring about change to their lives. Through the provision of support by the APN, the PNs in a forensic unit become confident and encouraged to implement what they have acquired in a real practical situation (see earlier in article results of step 1 defining and classifying central concepts as well as Table 2 thinking map of concepts).

The APN helps the PNs to be competent by encouraging them to master and internalise significant skills and qualities that will make it possible for them to use themselves as the therapeutic tool in the interactive-intervention process with the MHCUs in a forensic unit. Competencies that the PNs are facilitated to master are constructive self-concept, emotional control, effective communication skills, interpersonal skills, assertiveness skills and problem-solving skills.

Constructive self-concepts and identities are the central orientational devices with which PNs navigate and negotiate their everyday lives (Lofland et al. 2006:135). The PNs are assisted to gain self-confidence. Northouse (2013:24) states that self-confidence is the ability to be certain about one’s competencies and skills. It includes a sense of self-esteem and self-assurance and belief that one can make a difference in other people’s lives (Verderber, Verderber & Sellnow 2015:32).

Myburgh, Poggepoel and Du Plessis (2011:600) assert that emotional stability and ability to build and maintain healthy interpersonal relationships are essential in handling personal aggression. Ngambi (2013:45) adds that the hallmarks of self-control are openness to change, trustworthiness and integrity. A healthy and open dialogue depends on the leader staying attuned to others’ emotional states and controlling his or her own impulses to respond in ways that might not impair or restrict open communication (Manion 2011:117).

Effective communication plays a vital role in the management of aggression (Bimenyimana et al. 2016:6), and it leads to better interpersonal skills and also creates a better spirit of cooperation within the organisation (Konar 2011:160). Improvement in the emotional intelligence of nurses enhances their clinical competencies, including communication skills (Zhu et al. 2016:163). Remaining silent until the other person finishes speaking improves communication and strengthens relationships (Fine 2009:59).

Healthy interpersonal relationships can be attained by mastering interpersonal skills, which entails constructive communication, listening and responding and addressing interpersonal conflict (Naicker, Myburgh & Poggenpoel 2014:6) and Harrison and Hart (2008:23) add that genuineness enhances therapeutic interactions by allowing the helper to present himself or herself as a human being rather than as a role player. Empathetic attitudes allow people to see the world through the lens of other people, and active listening, respect and courtesy are essential pillars for high-quality relationship (Lapena-Monux et al. 2015:260).

Assertiveness is defined as the ability to have self-confidence about what you want and being able to communicate effectively about it (Perry 2007:206). Elder, Evans and Nizette (2010:433) add that some of the assertiveness skills that one can use in handling difficult situations include giving negative feedback or being confrontational. Myburgh and Poggenpoel (2009:447) believe that the attitude that one chooses when faced with a challenging situation is important, and one can choose to take a positive stand in an unaltered situation. Assertiveness results in self-confidence (Elder et al. 2010:433).

Problem-solving is focused on trying to solve immediate problems, which is viewed as a gap between ‘what is’ and ‘what should be’. Problem-solving and decision-making require critical thinking, which is a high-level cognitive process (Yoder-Wise 2009:93). Critical thinking includes being objective (Cottrell 2011:5) and taking a different perspective when looking at the same information. An open mind and the use of professional judgement are essential (Gopee & Galloway 2014:91).

In the termination phase, the measure for successful termination will be the extent to which the PNs have achieved the set objectives. Both the APN and PNs assess whether the PNs have been empowered through obtaining authority and competency in managing hostility by MHCUs in a forensic unit constructively. Psychiatric nurses are engaged in self-assessment and are encouraged to continue with self-assessment even after the termination phase, to maintain their level of competency.

#### Model evaluation

The evaluation of the model, by a panel of model development experts, was based on the criteria for model evaluation, as described in Chinn and Kramer (2011:195) as follows: clarity, simplicity, generality, accessibility and importance. The panel of model experts consisted of two professors, one associate professor and six lecturers with PhDs.

**Clarity:** Aspects that are looked into when determining how clear the model is are semantic clarity, semantic consistency, structural clarity and structural consistency, that is, understanding the intended theoretical meaning of the concepts and the relationships between the concepts within the theory. Initially, the concepts in the model lacked semantic clarity. Chinn and Kramer (2011:199) state that the clarity of the model is reduced by use of unrelated concepts in the model. Corrections and modifications were performed, for the model to meet the criteria.

**Simplicity:** The model appeared to be too busy, as the spiral was punctuated in all phases with arrows. Arrows pointing down, representing PNs withdrawing from the empowerment process, were removed. Modifications were performed and the model was found to be simple.

**Generality:** There was evidence that the model met this criterion. The model is a psychiatric nursing model proposed to be used in a forensic unit to facilitate the empowerment of PNs to manage hostility by MHCUs in a constructive manner. The scope of the concepts and the purpose within the model showed that the model has a broad scope of application.

**Accessibility:** Accessibility refers to the extent to which empiric indicators can be identified and the extent to which the purpose of the model can be achieved (Chinn & Kramer 2011:2003). The concepts of the model were found to be accessible, as they are linked to the empiric indicators in the forensic unit.

**Importance of the model:** There is evidence that the model meets the criterion. The model was found to have clinical significance, as it will bring in a significant change in psychiatric nursing practice, specifically in a forensic unit. The model is also of value in enhancing the personal and professional growth and development of PNs in the forensic unit.

## Limitations

The implementation of the model and also the evaluation of the implementation of the model have not been published yet.

## Recommendations

The findings of the study encouraged the researcher to make recommendations for psychiatric nursing practice, nursing education and nursing research. It has become apparent from the results of the study that participants benefitted from implementing the model in a forensic unit. The model could be used in all health settings where PNs encounter hostility by MHCUs. The nurse managers could also use the model to help subordinates who often display hostile behaviour towards them and co-workers. The model could benefit the families of MHCUs in the forensic unit, as well as the community and the society at large. In nursing education, the model could be integrated into the curriculum for the basic 4-year comprehensive nursing degree and diplomas in psychiatric nursing programmes. Lecturers could use the model when managing students who display hostility towards them and fellow students. It is recommended that further research be carried out by applying the model in different contexts.

## Original contribution of the study

The model serves as a solid foundation on which psychiatric students will be grounded during their training, which will enable them to be competent in managing hostile behaviour in a forensic unit. The model will help policymakers to formulate a policy regarding the managements of hostile behaviour by MHCUs in a forensic unit, as well as the policy to promote the mental health of PNs in a forensic unit. A mentally healthy PN will have a positive impact on the MHCUs, co-workers, the families of MHCUs and the community at large, and this will promote the integration of MHCUs into their families and community.

## Conclusion

In concluding this study, the researcher is of the opinion that the main purpose of the research has been achieved, that is, to describe, implement and evaluate a model as a frame of reference to facilitate the mental health of PNs in a forensic unit to constructively manage hostile behaviour by MHCUs. All steps of model development have been fully described, as well as the ethical consideration and trustworthiness. The PNs’ experiences on the implementation and evaluation will be discussed in a future article.
